# Barriers and facilitators to access mental health services among refugee women in high-income countries: a systematic review

**DOI:** 10.1186/s13643-022-01936-1

**Published:** 2022-04-06

**Authors:** Sarah DeSa, Akalewold T. Gebremeskel, Olumuyiwa Omonaiye, Sanni Yaya

**Affiliations:** 1 Interdisciplinary School of Health Sciences, University of Ottawa, Ottawa, ON Canada; 2 Faculty of Health Sciences, University of Ottawa, Ottawa, ON Canada; 3 School of International Development and Global Studies, University of Ottawa, Ottawa, ON Canada; 4 School of Nursing and Midwifery, Centre for Quality and Patient Safety Research, Institute for Health Transformation, Deakin University, Geelong, Australia; 5 Centre for Nursing and Midwifery Research, James Cook University, Townsville, Queensland Australia; 6 The George Institute for Global Health, Imperial College London, London, UK

**Keywords:** Mental health service, Refugee, Asylum-seekers, Women, Resettlement countries, Barriers, Challenges, Facilitators, Enablers, Access

## Abstract

**Background:**

Based on the Global Trends report from the United Nations High Commissioner for Refugee, in high-income countries, there are 2.7 refuges per 1000 national population, girls and women account for nearly 50% of this refuge population. In these high-income countries, compared with the general population refuge women have higher prevalence of mental illness. Thus, this review was conducted to examine the barriers to and facilitators of access to mental health services for refugee women in high-income countries for refugee resettlement.

**Methods:**

We searched MEDLINE, EMBASE, PsycINFO, and CINAHL databases for research articles written in English with qualitative component. The last search date was on March 14, 2020. A narrative synthesis was conducted to gather key synthesis evidence. Refugee women (aged 18 and older) that could receive mental health services were included. Men and women under non-refugee migrant legal status were excluded. Studies were evaluated studies using the Critical Appraisal Skills Programme (CASP) qualitative checklist.

**Results:**

Of the four databases searched, 1258 studies were identified with 12 meeting the inclusion criteria. Three studies were cross-sectional by design, eight studies used a qualitative approach and one studies used mixed approach. The major barriers identified were language barriers, stigmatization, and the need for culturally sensitive practices to encourage accessing mental health care within a religious and cultural context. There were several studies that indicated how gender roles and biological factors played a role in challenges relating to accessing mental health services. The major facilitators identified were service availability and awareness in resettlement countries, social support, and the resilience of refugee women to gain access to mental health services.

**Conclusion:**

This review revealed that socio-economic factors contributed to barriers and facilitators to accessing mental health among women refugees and asylum seekers. Addressing those social determinants of health can reduce barriers and enhance facilitators of access to mental health care for vulnerable populations like refugee women. A key limitation of the evidence in this review is that some data may be underreported or misreported due to the sensitive and highly stigmatizing nature of mental health issues among refugee populations.

**Systematic review registration:**

PROSPERO CRD42020180369

**Supplementary Information:**

The online version contains supplementary material available at 10.1186/s13643-022-01936-1.

## Background

Globally, in 2019, there were almost 71 million people forcibly displaced [1]. The experience of forceful migration has been acknowledged as a complex and traumatic life event that can significantly affect mental health in a negative way [2, 3]. Besides confronting the new changes in their environments, refugee populations are frequently overlooked when it comes to provision of health services, as well as access and delivery of mental health services [4]. According to the World Health Organization (WHO), “mental health is a state of well-being in which an individual realizes his or her own abilities, can cope with the normal stresses of life, can work productively and is able to contribute to his or her community” [4].

In relation to mental health promotion and mental health care plan for vulnerable populations such as refugees and migrants, the WHO in 2018 published a technical guidance report which provided a framework to addressing and promoting refugee’s social integration and platforms for overcoming barriers to accessing mental health care, facilitating engagement and utilization of services [5, 6].

About a third of people who have acquired refugee status live in high-income countries [7]. In the last several years, there has been a substantial increase in the number of refugees and asylum seekers seeking refugee and asylum status in major host and high-income resettlement countries [8, 9].. Worldwide, in 2019, about 64,000 refugees departed their home countries for resettlement in high-income countries [10]. With over 20,000 people arriving in the USA in 2019, which is about 24% increase from 2018, makes the country the highest recipient of arrivals of new refugees. Other high-income countries like Canada and the UK received 9031 and 5774 arrivals, a 17% and 1% increase from 2018, respectively. Other countries that have been on top 10 list of the United Nations High Commissioner for Refugee (UNHCR) in terms of resettlement of refugees in the past decade include Sweden with 4993 refugees arriving in 2019, Germany (4622), France (4544), and Australia (3464) [10]. With the increase of refugees in leading resettlement countries, it is crucial to understand the factors that influence access to the mental health care systems and the necessary interventions that can deal with the obstacles that refugees face in these resettlement countries [8, 9].

In each of the resettlement countries, the contexts of health service availability, accessibility, and refugee acceptance are different [8, 11]. The difference in cultural, political, economic, and social frameworks can play a considerable role in how a country prioritizes mental health service provision for refugee populations [11]. Additionally, in many of the host country the policies and entitlements relating to mental health care policies for refugee and asylum seekers are substantially different from the rest of the population [9, 12]. In most resettlement countries, there are systemic barriers that hinder refugees and asylum seekers from accessing mental health services. For example, the delivery of mental health care services in many of these high-income countries are often characterized by prolonged treatments which are not affordable because they are delivered by scarce and expensive mental health professionals [12]. Likewise, restrictive entry and integration policies have also been linked to poor migrant health outcomes in high-income countries [13]. Hence, it is important to understand how refugees are affected by migration policy in these high-income countries and its critical effect on access to mental health services.

By comparison to their male counter parts, evidence shows that there are risk factors that are specific to female refugees [14]. For instance, female Syrian refugees who arrived in Canada with trauma resulting from gender-based violence experienced in their country of origin who are now residing in a metropolitan city like Toronto, had many unmet health needs including mental health due primarily to lack of access to health services and in particular mental health care services [15–17]. Moreover, several studies have documented the psychological risks related to female refugees and the protection needs and challenges involved in managing mental health disorders in this vulnerable group of refugees [8, 18, 19]. The presence of psychological risks, such as having survived torture or serious violence, being a woman or girl at risk of abuse and exploitation, or facing persecution because of gender or sexual orientation, among many other devastating scenarios plays its intrinsic role in meeting eligibility of resettling in top resettlement countries [10]. Simply put, there should be an expectation of adequate mental health service provided to refugee women, especially considering that the selection criteria for refugee women that resettle in high-income countries are partially based on the premise of trauma exposure [10].

To provide care and mental health services to refugee women, identifying and addressing barriers and facilitators that directly impact access, use, and utilization of mental health services need to be understood [9, 20, 21]. Studies have identified social and structural constructs that exist as barriers to the ease and use of mental health services among vulnerable populations such as refugee women; these barriers include but not limited to the following scheduling or restrictive timing, linguistic barriers, low social status, discrimination, and stigmatization [20–25].

It is acknowledged that there is a need for mental health services to be accessible and acceptable within resettlement countries and should be consistent with the needs and difficulties of refugees.

While there are numerous studies that have examined the barriers to accessing mental health services, there is a paucity of studies that have identified the facilitators and enablers of access to mental health care among refugee population, including the delivery of culturally sensitive intervention [25], and the provision of gender concordant services [14, 16, 26]. Although, there is large body of literature that have examined the barriers that influence mental health, there is an absence of systematic reviews that have specifically examined the evidence on barriers to and facilitators of access to mental health services for women refugees and asylum seekers in high-income countries [8, 27]. In this context, the aim of this review is to examine existing barriers and facilitators to accessing mental health services for refugee women in leading high-income countries for refugee resettlement.

## Methods

This review aimed to identify and collect qualitative data from eligible studies conducted in high-income countries for refugee resettlement.

### Protocol registration and reporting

The present review has been registered within the PROSPERO database (registration number CRD42020180369). The published protocol for this systematic review [28] was written according to the Preferred Reporting Items for Systematic Review and Meta-analysis Protocols (PRISMA-P) guideline for reporting systematic reviews [29]. This review was conducted as per the Cochrane Collaboration Handbook of Systematic Reviews [30] and the findings were reported in accordance with the reporting guidance provided in the PRISMAs statement [29] (see Additional file 3 for details) and the Enhancing Transparency in Reporting the Synthesis of Qualitative Research (ENTREQ) statement [31].

### Inclusion and exclusion criteria

Eligible studies comprised of original research, peer-reviewed articles that had a qualitative component (i.e., qualitative, mixed-, or multi-method studies) written in English language.

The included studies involved refugee women (aged 18 and older) that could receive mental health services. Furthermore, studies conducted for both sexes were considered for inclusion, but only data for women were extracted. The eligible studies were conducted in leading high-income countries for refugee resettlement based on data from 2009 to 2019 in the UNHCR’s Global resettlement needs reports [10] and involved one or more type of usual standard mental health service for refugee women, including abuse support, addiction support, counselling, crisis support, psychiatric and psychological assessments and treatments, and support groups. Studies reporting data from countries outside of the UNHCR’s leading resettlement countries from the past decade were excluded. The published protocol manuscript provides more details on the predefined eligibility criteria for this review [28]. Furthermore, Additional file 1 also contains detailed information on the inclusion and exclusion criteria.

### Information sources and search methods

The primary source of literature was a structured search of major electronic databases (from the date of the inception of the database): MEDLINE (Ovid), EMBASE, PsycINFO, and CINAHL. The search strategies comprised of the following stages. First, a search of MEDLINE (Ovid) to identify relevant keywords contained in the title, abstract, and subject descriptors. Second, we identified the synonyms and related terms for searches in EMBASE, PsycINFO, and CINAHL. In addition, we performed hand-searching of the reference lists of included studies, relevant reviews, or other relevant documents. The searches included a broad range of MeSH terms and keywords related to mental health services, accessibility, refugee, asylum seeker, women/female, and qualitative research. A final search was conducted on March 14, 2020, and a draft search strategy within multiple databases is provided in Additional file 2. MeSH terms related to mental illness were not included, as this review focused on accessing mental health services and not necessarily the presence of mental illness (see Additional file 2 for more details).

### Selection of studies

Citations were imported into the Zotero citation management software and uploaded in a zip file. The articles retrieved from searches within each database were uploaded into the Covidence article management system and screened by two authors within the Covidence database for their relevance and eligibility to the review. Two reviewers (AG and SD) independently screened the titles and abstracts according to a pre-defined inclusion criteria checklist and excluded unrelated ones. Disagreements were resolved through discussion with SY and OO. This included title and abstract screening, followed by full-text screening against the eligibility criteria for studies deemed potentially eligible. Disagreements were settled through discussion. The PRISMA (Preferred Reporting Items for Systematic Review and Meta-Analyses) flowchart was used to document the selection process [29].

### Data extraction and management

Following full-text screening, data was independently extracted from the retrieved eligible studies by two of the reviewers (ATG and SD). Disagreements were settled through discussion with SY and OO. The authors adapted a data collection document based on the needs of the review from a standardized data extraction form by the Cochrane Handbook [30]. The data that was extracted included all details specific to the review question, fulfilling the requirements for a narrative synthesis.

### Assessment of risk of bias in included studies

A critical appraisal of included studies was conducted by two reviewers independently. All disagreements were resolved through discussion or consultation with the third and fourth reviewer as needed. Results from the appraisal were summarized narratively to highlight strengths and limitations within and across studies. The reviewers evaluated the studies using the appropriate Critical Appraisal Skills Programme (CASP) checklist [32] (see Additional file 4 for details). The included studies were assigned an overall score of ‘high’ (9–10), ‘moderate’ (7.5–9) or ‘low’ (less than 7.5) for overall quality. Studies were not excluded or weighted based on the quality of the reporting assessment, instead, the results of the appraisal were used to inform data interpretation and help confirm the validity of review findings and conclusions. Therefore, seven studies were high quality (≥ 85%), five studies were moderate quality (75–85%), and none was of low quality (≤ 75%).

### Certainty of evidence

The GRADE-CERQual (“Confidence in the Evidence from Reviews of Qualitative research”) approach was applied to assess and summarize confidence in key findings [33]. This provided overall confidence in each of the key findings. Two reviewers independently assessed the certainty of evidence using the GRADE-CERQual approach [33]. Disagreements were resolved through discussion. Results were presented in GRADE-CERQual summary of qualitative findings tables [33].

### Data synthesis

Evidence table of an overall description of the studies, data from each paper that provided details of study characteristics, context, participant immigration status, outcomes, and conclusion. A textual narrative synthesis was conducted, a method that was ideal for synthesizing evidence from a wide range of research questions and study designs with qualitative, mixed- or multi-method approaches, as the emphasis is on an interpretive synthesis of the narrative findings of research [34]. The data was described in a narrative synthesis, grouped by study type, participant characteristics, review objective, and outcome (see Additional file 5 for details). Accordingly, barriers and facilitators of mental health services for refugee women in high-income countries were identified and summarized.

## Results

### Search result

**Fig. 1:**
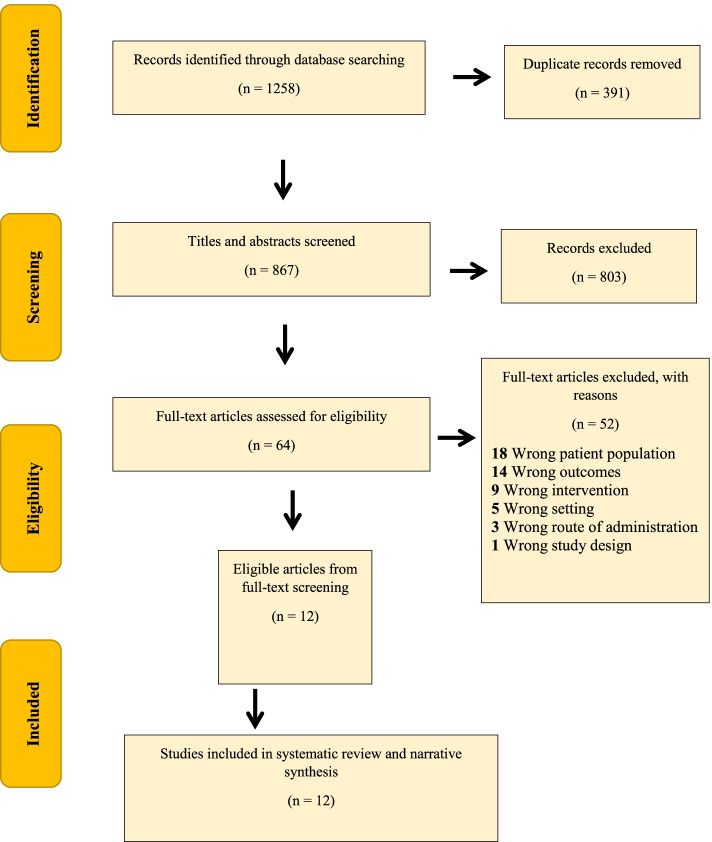
PRISMA Flowchart

The reviewers identified 1258 records through database searches. After removing duplicates and conducting title and abstract screening and full text screening, there were 12 records that remained for inclusion within this systematic review. Details of the selection process and the reason for exclusion of excluded articles are provided on PRISMA flowchart (see Fig. 1 for details).

### Study characteristics

Three studies were cross-sectional by design [35–37], eight studies used a qualitative approach [38–45], and one studies used mixed approach [46]. Accordingly, seven studies were conducted in North America, with five being conducted in Canada [38–40, 42, 46] and two was conducted in the USA [36, 45]. Four studies were conducted in Australia [37, 41, 43, 44], and one study was carried out in Europe (UK) [35] (see Table 1 for details).

**Table 1:** Study and participant characteristics

Author	Year	Study setting	Study design	Participant characteristics	Immigration status	Participant country of origin	Type of usual standard mental health services for refugee women
Whittaker, et al.	2005	UK	Cross-sectional	Female refugees born in north Somalia, entered UK as a child or adolescent living in England for over 1 year	Asylum-seeker; refugees	Somalia	Doctors, counsellors, “shrinks”, psychologists, bereavement groups, telephone help lines, homecare
Wong, et al.	2006	USA	Cross-sectional	Refugees from Cambodian origin between 35 and 75 years old	Refugees	Cambodian	Mental health care services
Donnelly, et al.	2011	Canada	Qualitative	Refugee and migrant women living with mental illness.	Refugees	China and Sudan	Mental health care services
Drummond, et al.	2011	Australia	Cross-sectional	Refugee women aged between 20 and 67 years from Liberia or Sierra Leone who have lived in Australia for 6 months to 5 years.	Refugees	Liberia, Sierra Leone	Health care services and mental health services
O'Mahony, et al.	2013	Canada	Qualitative	Over 18 years old, non- European women with immigrant or refugee s status, living in Canada	Refugees	Costa Rica	post-partum depression mental health services
Piwowarczyk, et al.	2014	USA	Qualitative	Refugee women from Congolese or Somalian backgrounds, above the age of 18 years old. (Age range 18–59)	Refugees	Somalia and Congolese	Mental health services
Ahmed, et al.	2017	Canada	Mixed-methods	Pregnant or within 1 year of giving birth, admitted to Canada as government or privately sponsored refugees, able to speak English and Arabic. (Age range:20 to 37 years)	Refugees	Saskatoon, Canada	Natal and antenatal mental health care
Clark N.	2018	Canada	Qualitative	Karen women receiving frontline health care services (Age range: 26–60).	Refugees	Karen speaking; Burmese, and Thailand	Mental health services
Smith, et al.	2019	Australia	Qualitative	Adult and youth former refugee and essential service providers residing in Launceston.	Refugees	Afghanistan, Bhutan, Burma, Sierra Leone, Sudan and Iran	Mental health essential services
Willey, et al.	2019	Australia	Qualitative	Refugees that are in the last stages of pregnancy or post-natal pregnancy care, living in a suburb in Melbourne.	Refugees	Burmese and Dari- Afghan, Indian, Vietnamese refugee women	Perinatal mental health screening
Babatunde Sowole, et al.	2020	Australia	Qualitative	Women aged 18 years and older who are fluent in English living in Australia for over 12 months.	Asylum-seeker; refugees	West Africa	Mental health provision
Tulli, et al.	2020	Canada	Qualitative	Immigrant and refugee mothers living in Edmonton, Canada, and have children living in Canada. (Age range:18–50)	Refugees	Sudan, Syria, Colombia	Pediatric and immigrant mental health care

### Participant characteristics

Participant were women either of refugee or asylum seeker status originating from Africa (Somalia, Nigeria, Gambia, Mali, Eritrea, Sudan, Sierra Leone, Congo, Liberia) Asia (Burma, Thailand, Pakistan, Vietnam, India, China, Bhutan) Middle East (Syria, Afghanistan, Iran, Iraq), and South America (Costa Rica, Colombia). Most participants sought general mental health services and three out of twelve studies reported that participants sought mental health services related to antenatal care such as post-partum depression. The age range of participants in this review was 18–75 years (see Table 1 for details).

### Barriers to accessing mental health services for refugee women in leading high-income countries for refugee resettlement

All of the studies (12/12) reported barriers to accessing mental health services among women refugees in top resettlement countries. There were three main barriers that were commonly mentioned in all the studies [35–46]. Those are language barrier, stigmatization and lack of culturally appropriate resources.

### Language

The challenges to accessing mental health services included the importance of linguistics and language barriers between participants and their health care provider [36, 38, 40–46]. Participants in these studies expressed the heavy reliance on interpreters, they specifically voiced concern for lack of funding of language services and lack of available interpreters in the health care system [44]. Studies showed that because of the limited grasp of English, participants expressed difficulty in understanding the health system, and medical terminology; these factors hindered appointment-scheduling and led to missed clinical appointments [40, 42, 44]. Similarly, studies have shown that language barriers also interfere with participants benefiting from available mental health counselling services and community-based health programs [40]. Participants spoke about using family members as interpreters in situations where there was a lack of available interpreters. However, conflicts of interest arose when family members often failed to translate exact messages or were sometimes the subjects of the issues being disclosed to mental health care providers [40]. This conflict of interest and lack of confidentiality was an issue of utmost importance to participants and using lay interpreters that sometimes also had limited English literacy and communication skills [40].

### Stigmatization

Another barrier to accessing mental health was the stigmatization experienced by refugee women seeking help for mental health care among family and their community [35–37, 41–43, 45, 46]. Mental health stigma was a theme most recognized among respondents across the various cultural groups of refugees. Apart from the stigma associated with having mental health issues, stigma was also apparent when trying to engage in help-seeking behavior. The notion of stigma extended to a varying degree of relationships of participants across the cultural groups and across studies. Participants most reported perceptions of stigma from within and outside their community [35–37, 41–43, 45, 46], but also included the fear of being stigmatized by their spouses. An example, are the words of women from Piwowarczyk, et al. 2014 study [45], which stated:

“If you go, your husband or boyfriend will discourage you. You don’t go to strangers to talk about something shameful” (Congolese woman, 18–25 years). “Most would not go to a psychiatrist, don’t want to talk to outsiders” (Somali woman, 18–25 years).

“[husbands think] The same thing (as community responses to seeking help for mental health), that ‘she’s losing it’” (Somali woman, 25–36 years).

These types of similar narratives revealed that people may not necessarily be inclined to disclose to others about their suffering, which can result in negative consequences to their quality of life [37, 45].

### Lack of culturally appropriate resources

Another barrier to accessing mental health care included the need of culturally appropriate resources when accessing mental health services. The lack of trust in western medicine and the use of medication promoted by doctors in resettlement countries had some participants hesitant to adopt this form of treatment due to its cultural significance [40]. Additionally, concerns about confidentiality, privacy from the use of lay interpreters, and concerns of accuracy from the use of health care workers proved to be big obstacles in accessing mental health services [43]. Through the inclusion of culturally sensitive care, perceptions and expressions of mental health needs can be relayed within an ethnic or cultural context to health care professionals [43]. Participants voiced concerns that health systems in resettlement countries, like Canada, are not sensitive to the unique cultural needs of immigrants and refugees thus resulting in further isolation within these populations [40]. Culturally sensitive care included the need for practices wrapped in religious and cultural contexts [35, 40, 43, 45]. Spiritual practices not only were identified as a source of strength and hope, but also deterred women from accessing western biomedical treatment [40]. Participants explained misunderstandings related to treatments which adhered to a westernized medical model, failing to incorporate culture and belief system of the people [44]. Concepts like *Zar* possessions in Somalia were a concept of spirit taking control over community members [35]. They believed *Zar* spiritual possessions could be explained by anger, unusual behavior, nightmares, pains, and even pregnancy. Participants struggled to hold traditional and religious beliefs with scientific and medical ones [35]. A refugee participant from Whittaker, et al. 2005 study [35] stated:

“Religious leaders... can do certain spiritual things to kind of release her from whatever’s possessing her… So, I’d just take her, I don’t know, to a mosque or something.” (Monique)

### Gender roles

The role of gender and the associated consequential biological factors play a role on how refugee women access mental health service [37–43, 45, 46]. The variety of ways gender role affects mental health access, specifically the contribution to the family dynamic and the unique experience of being a woman and dealing with gender specific concerns such as post-partum depression [39, 41, 46]. For example, refugee women shouldered the responsibility of childcare and diminished the priority to take personal care of themselves such as accessing mental health care [38, 42]. Furthermore, findings showed a gender hierarchy within relationships that portrayed male domination and control affecting women’s health status and timely access to mental health services, increasing their social vulnerability in a dependent situation [38–40]. O’Mahony, et al. 2013 indicated that participants clearly identified that behavior of their partner or spouse was a contributing factor to their post-partum depression. New immigrant and refugee mothers voiced that they found themselves in a powerless and generally dependent position that left them vulnerable to abuse [39].

### Facilitators to accessing mental health services for refugee women in leading high-income countries for refugee resettlement

This review found that seven studies reported that there are facilitators to accessing mental health services among women refugees in top resettlement countries [35, 38, 40–42, 45, 46]. Those are service availability and awareness, social support, and resilience factors.

### Service availability/awareness of mental health services

Service availability and awareness of mental health services also facilitated ease of access depending on the resettlement country and the national health and immigration policies that were set in place [35, 38, 41, 42]. Participants expressed their gratitude towards their hosting resettlement country for providing mental health services that they were eligible for. Equally importantly, participants mentioned strong health promotion of mental health resources to aid in accessing mental health services offered [35, 38, 41, 42]. The availability and awareness of services included the provision of mental health and mental illness prevention and treatment care at the primary health care level, but also refugee resettlement agencies that provided a network of caseworkers and mental health counselling services [38]. These types of programs that made mental health services available to refugees enabled ease in access in continued mental health care [35, 38]. Donnelly et al. 2011 showed that participants talked about the awareness of mental health and well-being among refugee women is a positive change in the community. The study indicated the having written resources in their own language and making them available in public places like community centers.

### Social support

The importance of social support was identified as key facilitator among respondents in accessing mental health services [38, 40, 41, 45, 46]. Support from spouses, family, and the community provided varying degrees of support for easing mental health illnesses like stress, depression and anxiety in everyday life [38, 40, 41, 45, 46]. Social support also provided encouragement in help-seeking behavior for mental health services and improved self-efficacy and autonomy of participants. An example of a participant’s experience of social support, related to a post-partum depression experience is taken from Ahmed et al. 2017 study [46] stating:

Reem: “It should be like we can go and visit them, or the family can come and visit us. This way we would not feel like a bird in a cage or imprisoned…. This is the most important and critical issue here. This is what can cause depression.”

Joud: “You feel that you aren’t alone, and you will feel that someone is standing beside you.… Like when you are going to give birth, you really need your mother, your aunt or somebody.”

Additionally, participants identified that knowledge and awareness of mental illness by family members were important in continuing access to proper treatment and professional interventions, making mental health education imperative.

### Resilience

There were some studies that identified the importance of autonomy of refugee women [38, 40, 43]. The theme of resilience among refugee women was consistent across all studies but was explicitly outlined as a feature that exemplified the resilience strength and determination of the women [38, 40]. The participants sought assistance and employed self-care strategies to deal with mental health problems within the context of limited resources [40]. The concept of resilience and coping resonated among female participants that voiced their independence and high standard of self-efficacy that enabled help-seeking behavior and eased accessing mental health services [43]. Though refugee women recalled sad stories of trauma and sorrow, participants also showed strong and liberating values when it came to taking control of their lives and being determined to survive in a new resettlement country. Such admirable traits exhibited, were shown as empowering refugee women to access the mental health care they needed. Ahmed, et al. 2017 showed participants mention that partaking in exercises like walking and swimming helped their mental health [46]. Participants exemplified resilience by taking control of their life and engaging in activities that facilitate sound mental health care [46]. These mental health care services included support programs and gendered recreational group classes that helped refugee women form social connections and build social networks to make them feel supported [46].

## Discussion

There is a large body of evidence that pertains to the barriers and facilitators which refugee women face when accessing mental health services in resettlement countries. This systematic review summarizes the evidence regarding mental health care barrier and facilitator study characteristics, context, and participant’s sex and age, to address the experiences faced by refugee women when accessing mental health services in leading high-income resettlement countries. The findings of this research may be applied to enhance existing mental health service access for refugee women in high-income countries.

The review revealed the prevalence of barriers to accessing mental health services, the most common being stigmatization of mental health and mental health-seeking behavior [35–37, 41–43, 46]. Stigma is a theme that participants commonly discussed, and often related their understanding to how an individual and society views their mental health [46–51]. Participants feared how mental health will be linked to their country of origin, families, and resettlement in immigrant communities [46]. When interviewed, refugee women mentioned the fear of disapproval of ethnic community members, family, and friends, as well as physical and emotional abuse from their partners as a consequence of seeking help or divulging mental health concerns [48, 49]. Patterns of stigma and discrimination can be multi-fold, based on social identity [49]. Many studies support the notion that stigma and negative attitudes towards mental health and mental health services users play an influential role across refugee population and trauma survivors [50–53]. Shame is known to impede help-seeking combined with the embarrassment among sexual violence survivors [54].

The role of gender is an imperative component of this review, as almost all the included studies wrap the involvement of barriers and facilitators in accessing mental health as it pertains to the female refugee experience [35, 37–43, 45, 46]. Women face barriers that are specific to biological factors because of their disposition to postpartum depression, a barrier that is non-existent for their male counterparts [41, 43, 48, 55–57]. As such, the need for sufficient natal and antenatal mental health care is a concern that is worsened among refugee women predisposed to trauma conflict and violence [41, 43, 48, 55–57]. Additionally, based on the findings, some participants suggested that a gendered hierarchy existed within some relationships, including a dependency on their husbands. Characteristics of social vulnerability were parallel to those that were described by refugee women participants [58–61]. These characteristics included not having other family in their resettlement country, not being financially independent, having an undocumented status, and lack of education that perpetuated intimate partner violence [60].

Social support from the spouse, family, or community offered to refugee women enabled access and promoted help-seeking behavior for mental health care [38, 40, 41, 45, 46]. While there are less studies that looked into facilitators of accessing mental health services, social support was a reoccurring theme voice by participants when discussing factors that contributed to accessing mental health care [38, 40, 41, 45, 46]. Similarly, several studies have shown the significance that social support provides to accessing mental health services among refugee populations [45]. Somali populations have supported their community members to provide social support through providing advice about treatment and coping behavior, financial aid for seeking treatment, and religious guidance [52]. Friends and family were known as important gatekeepers to mental health service utilization and access, as they encourage women to seek professional treatment [61, 62].

### Implications

This review revealed socio-economic factors as potential contributing facilitators and barriers to access mental health among refugees and asylum seekers. Policymakers would benefit in addressing social determinants of health in order to remedy the concerns addressed by participants when accessing mental health services [63, 64]. Increasing education and financial stability related to job and housing security cannot only reduce added stresses of everyday life [63, 64], but also can reduce social vulnerability of refugee women. Policy recommendations to address these kinds of social determinants of health can reduce barriers and enhance facilitators of access for mental health care to vulnerable populations like refugee women.

Additional benefits of this review aim to reshape the view of refugee women from the associated stigmatization related to mental health to a narrative that showcases the resilience and strength of refugee women who are determined to access mental health services. Based on the body of evidence from this review, the undeniable relationship between the availability of social support and access to mental health services. This review cannot only inform community outreach and public health programs to include and involve a greater focus on community engagement and social support or outreach to reduce isolation and stressors, but also to encourage community members in refugee populations and other social setting to address and promote mental health care.

The review findings suggest the need for further research on this topic given the potential significance of the findings on refugee and asylum seeker women mental health.

## Limitations

This review gathers qualitative data to examine existing barriers and facilitators to accessing mental health services for refugee women in leading resettlement countries. There are several limitations of our systematic review methods. There is an exclusion of research published in languages other than English, which can result in the exclusion of valuable data. Additionally, some data may be unrepresented, underreported or misreported due to the sensitive and highly stigmatizing nature of mental health issues among refugee populations. This may result in publication bias and methodological quality issues. Lastly, there were many studies in this review that made use of a cross-sectional research design. Factors that are related to the length of time living in a resettlement country plays a role in the influence on awareness and utilization of mental health services, therefore the adoption of a longitudinal research design may be most appropriate for future research to assess the barriers and facilitators for accessing mental health services over time.

## Conclusion

This systematic review examined the facilitators and barriers to access mental health services among refugee women. Evidence suggests that stigmatization of mental health from within the refugee population is a major barrier for women refugee mental health-seeking behavior. One important facilitator of mental health services among women refugee is the strong health promotion of mental health resources in the countries of resettlement which aided access to mental health services. It will hope to inform community outreach and public health programs to include promote and encourage accessing mental health services among refugee women populations.

## Supplementary Information

**Additional file 1.** Study Selection Criteria.

**Additional file 2.** Search Strategy.

**Additional file 3.** PRISMA Checklist.

**Additional file 4.** CASP Checklist.

**Additional file 5.** Narrative Synthesis.

## Abbreviations

CASP: Critical Appraisal Skills Programme
PRISMA-P: Preferred Reporting Items for Systematic Review and Meta-analysis Protocols
PROSPERO: International Prospective Register of Systematic Reviews
UNHRC: United Nation High Commissioner for Refugee
WHO: World Health Organization
