# Supplementary material for: Barriers and facilitators to access mental health services among refugee women in high-income countries: a systematic review
Source: Syst Rev. 2022 Apr 6;11:62. doi: 10.1186/s13643-022-01936-1 (PMC8985267; doi:10.1186/s13643-022-01936-1)
Supplement: Supplementary file 1 — Additional file 1. Study Selection Criteria. [file 13643_2022_1936_MOESM1_ESM.docx]

Inclusion and Exclusion Criteria

|  | **Inclusion Criteria** | **Exclusion Criteria** |
| --- | --- | --- |
| **Participants** | - Refugee women/females in leading resettlement countries. - Refugee women aged 18 and older that receive mental health services. - Refugee women data provided within gender comparative studies. | - Refugee men in resettlement countries that receive mental health services. - Men and women under non-refugee/asylum seeker/ displaced migrant legal status. |
| **Context** | - Involves one or more type of usual standard mental health services for refugee women, including abuse support, addiction support, counselling, crisis support, psychiatric and psychological assessments and treatments, and support groups. | - Usage or need for non-mental health services for refugee women, including other health services unrelated to mental health treatment or prevention. |
| **Comparison / Control Group** | - No comparison group for this study. |  |
| **Outcome of Interest** | - To present barriers or challenges, facilitators or enablers, related to accessing mental health services in leading resettlement countries. |  |
| **Setting** | - Leading high-income countries based on data from 2009-2019 in the UNHCR’s Global resettlement needs reports. - Included resettlement countries include: - North America: Canada and   United States of America   - Europe: Belgium, Denmark, Finland, France, Germany, Netherlands, Norway, Switzerland, Sweden and United Kingdom - Oceania: Australia and New Zealand | - Countries not included as a leading resettlement countries from the past 10 years. |
| **Study Design** | - Qualitative, mixed- or multi-methods studies. - Peer reviewed full-text research papers, published in English. | - Reviews, editorials and commentaries, discussions, theses or dissertations and other gray literature. - Articles published in any other languages other than English. |
