# Supplementary material for: Barriers and facilitators to access mental health services among refugee women in high-income countries: a systematic review
Source: Syst Rev. 2022 Apr 6;11:62. doi: 10.1186/s13643-022-01936-1 (PMC8985267; doi:10.1186/s13643-022-01936-1)
Supplement: Supplementary file 4 — Additional file 4. CASP Checklist. [file 13643_2022_1936_MOESM4_ESM.docx]

**Additional file 4: CASP Checklist**

|  | Whittaker, et al.,2005 | Wong, et al., 2006 | Donnelly, et al., 2011 | Drummond, et al., 2011 | OMahony, et al., 2013 | Piwowarczyk, et al., 2014 | Ahmed, et al., 2017 | Clark N., 2018 | Smith, et al., 2019 | Willey, et al., 2019 | Babatunde Sowole, et al., 2020 | Tulli, et al., 2020 |
| --- | --- | --- | --- | --- | --- | --- | --- | --- | --- | --- | --- | --- |
| Was there a clear statement of the aims of the research? | Y | Y | Y | Y | Y | Y | Y | Y | Y | Y | Y | Y |
| Is a qualitative methodology appropriate? | Y | Y | Y | Y | Y | Y | Y | Y | Y | Y | Y | Y |
| Was the research design appropriate to address the aims of the research? | Y | Y | Y | Y | Y | Y | Y | Y | Y | Y | Y | Y |
| Was the recruitment strategy appropriate to the aims of the research? | Y | Y | Y | Y | Y | Y | Y | Y | Y | Y | Y | Y |
| Was the data collected in a way that addressed the research issue? | Y | Y | Y | Y | Y | Y | Y | Y | Y | Y | Y | Y |
| Has the relationship between researcher and participants been adequately considered? | Y | Y | Y | Y | Y | Y | Y | Y | N | Y | Y | Y |
| Have ethical issues been taken into consideration? | Y | Y | Y | Y | Y | Y | Y | Y | Y | Y | Y | Y |
| Was the data analysis sufficiently rigorous? | N | Y | Y | Y | Y | Y | Y | Y | N | Y | N | Y |
| Is there a clear statement of findings? | ? | Y | Y | Y | Y | Y | Y | Y | Y | Y | N | Y |
| Is the research valuable? | Y | Y | Y | Y | Y | Y | Y | Y | Y | Y | Y | Y |
| Overall Score | 8 | 9 | 9.5 | 9 | 9.5 | 8.5 | 9 | 8.5 | 8.5 | 9 | 8 | 9.5 |
