# Supplementary material for: Barriers and facilitators to access mental health services among refugee women in high-income countries: a systematic review
Source: Syst Rev. 2022 Apr 6;11:62. doi: 10.1186/s13643-022-01936-1 (PMC8985267; doi:10.1186/s13643-022-01936-1)
Supplement: Supplementary file 5 — Additional file 5. Narrative Synthesis. [file 13643_2022_1936_MOESM5_ESM.docx]

**Additional File 5: Narrative Synthesis**

Whittaker et al., 2005 used a cross- sectional approach to investigate how young Somalian refugee and asylum-seekers perceive psychological well-being [35]. Using semi-structured personal and group interviews of female refugees born in north Somalia, Whittaker et al., focused on participants that entered the United Kingdom as children or adolescents and lived in England for at least 1 year [35]. They examined the access to doctors, counsellors, psychologists, bereavement groups, telephone help lines and homecare. The study identified facilitators to accessing mental health services among refugee women such as having mental health services available to refugee populations, as well as, resilience factors (like self-esteem and efficacy), religious and cultural social support [35]. Conversely, the study identified barriers of accessing mental health services such as confidentiality, trust in western treatments, fear of judgement and stigma [35].

Clark, 2018 uses their ethnographic research to examine Karen refugee women's experiences and the social structural factors which facilitated or challenged community capacity to support mental health during resettlement [36]. Using in-depth interviews and focus groups, this study assessed the access to standard mental health services in Canada among Karen speaking refugees originating from Thailand and Burma [36]. The study found that dependency on education and educated family facilitated access to health services [36]. Other facilitators included class position of Karen families as well as trust in interpreters, whereas barriers to accessing mental health services included lack of appropriate supports made them feel constraints [36]. Additionally, participants mentioned there was a lack of available interpreters; lack of funding for language services; lower health literacy and education available; the need for structural reform; and being unable to navigate health care resources [36].

O'Mahony et al, 2013 explored how cultural, social, political, historical, and economic factors intersect with race gender and class to influence the ways in which refugee women seek and access mental health services through a critical ethnography [37]. The study used purposive sampling to collect data through personal interviews of non-European immigrant or refugee women, that were 18 years old or old living in Canada with post-partum depression [37]. The findings of this study highlighted barriers of mental health access among refugee and immigrant women from Costa Rica. Barriers to mental health access included the importance of gender roles within the family dynamics and how a precarious immigration status can be related to mental health of immigrants and refugees through the consequence of no status [37]. Another barrier mentioned by participants were the dependency on others to translate and navigate the corrective routes to mental health care [37].

Donnelly et al., 2011 used a descriptive exploratory qualitative study to increase understanding of the mental health care experiences of immigrant and refugee women by inquiring about information regarding factors that either support or inhibit coping [38]. Using in-depth interviews, refugee women living with mental illness that were primarily of Chinese and Sudanese origin, living in Canada were shared their experiences of enablers to accessing mental health services [38]. Facilitators included informal support systems, self-care practices, education, knowledge and awareness of mental illness of family members, trust, rapport and faith in health care providers [38]. Conversely, participants also identified barriers accessing mental health services such as lack of awareness of mental health services available and lack of appropriate and culturally sensitive mental health services [38].

Willey et al., 2019 adopted a phenomenological study design to determine if a perinatal mental health screening program is feasible for refugee women [39]. The study included interview and focus groups to recruit refugee women that were in the last stages of pregnancy or post-natal care, living in a suburb in Melbourne, Australia [39]. Participants originated from Burma, Afghanistan, India, and Vietnam, and used perinatal mental health screening. Findings from this study expanded on facilitators of access mental health services such as family support and emotional well-being [39]. However, there were barriers also identified such as stigma, and language barriers between health professionals and participants [39].

Tulli et al., 2020 uses a qualitative descriptive design to explore immigrant and refugee mothers’ perceptions of barriers and facilitators to accessing mental health for their children [40]. Semi-structured interviews were conducted with Sudanese, Syrian and Colombian immigrant and refugee mothers living in Edmonton, Canada who have children also living in Canada [40]. Participants were asked about their perception on the quality of mental health care and the adequacy of access to mental health care for their children [40]. This study found that there were enabling factor related to accessing mental health services such as availability of free services offered, high levels of education that contributed to ease in access, as well as, the variety of services and treatments offered [40]. The participants also identified challenges to accessing mental health care such as financial strain, feeling unheard during service intake, lack of information, racism and discrimination, stigma, and language barriers [40].

Ahmed et al., 2017 used a qualitative driven mixed method study to understand the experience of Syrian refugee women dealing with maternal depression in Canada [41]. The researched used a written questionnaire and focus groups of refugee women who were either pregnant or had given birth within the year [41]. Eligible participants were required to be admitted to Canada through government or private sponsorship and be able to speak English or Arabic and were assessed for their natal and antenatal mental health care [41]. The study found that there were facilitators and barriers to accessing mental health services, including enablers such as strong social support including emotional support from the family [41]. Additionally, government support with the provision of mental health services, financial and social programs of support to minimize excessive stress [41]. Conversely, stigma of mental health, privacy and confidentiality concerns, and language difficulties were common barriers that were highlighted during discussions about accessing mental health [41].

Babatunde‐Sowole et al., 2020 used a quality inquiry to explore and create awareness about west-African women's resilience prior to migration and post migration needs of support for trauma- informed care [42]. Using purposive snowball sampling, researchers conducted interviews with participants living in Sydney, Australia who originated from various parts of West Africa [42]. The study illustrated factors that enabled access to the provision of mental health care, such as resilience in adverse life situations within their host country [42]. On the other hand, participants also reiterated challenges in access mental health services like language barriers, distrust in host countries and western practices, experiences with xenophobia, and factors of job security related to immigration status that exacerbated stress and anxiety [42]. Stories of being unable to disclose mental health concerns related to physical and sexual violence epitomized some women’s experiences [42].

Smith et al., 2019 used a phenomenological approach to their study in order to examine the resettlement experience of former refugees living in regional Australia, focusing on mental health and support services including barriers to access [43]. The study looked at the experience of former refugee youth and adults originating from Afghanistan, Bhutan, Burma, Sierra Leone, Sudan, and Iran that reside in Launceston, Tasmania [43]. Using semi-structured interviews and focus groups, researchers identified barriers to accessing mental health services such as language and the se of interpreters, the lack of culturally informed practices, and trauma informed care [43].

Wong et al., 2006 conducted a cross-sectional study to asses structural and cultural barriers to mental health care among refugees from Cambodia living in the USA [44]. The researchers conducted interviews and surveys with Cambodian women between 35-75 years old to find out how they can access and use mental health services [44]. The study identified barriers to accessing mental health such as stigma, lack of culturally sensitive care, language barriers, financial barriers, physical barriers like transportation to facilities [44].

Piwowarczyk et al., 2014 used a qualitative inquiry approach to examine both concepts of mental illness in addition to attitudes and beliefs about treatment as well as potential barriers to accessing mental health services [45]. Refugee women from Congolese or Somalian backgrounds, living in Boston, USA, above the age of 18 years old were convenience sampled for focus groups [45]. The outcomes of the study identified barriers to accessing mental health services such as the need for utilizing traditional methods of healing, lack of family support, stigma, financial strain, and a distrust or misunderstanding in western mental health services [45].

Drummond et al., 2011 used a cross- sectional study to identify barriers to accessing health care services among West African refugee women in Perth, Australia [46]. Researchers recruited refugee women between 20-67 years old from West Africa, who have lived in Australia between 6 months to 5 years [46]. Through interviews, refugee women from Liberia and Sierra Leone relayed experiences of barriers faced when accessing health services and mental health care, which included stigma, distrust in western medicine, distrust in medication, trying to cope alone, lack of awareness of services available, financial strain, and transportation as a physical barrier of access [46].
